# Supplementary material for: Vitamin D Modified DSS-Induced Colitis in Mice via STING Signaling Pathway
Source: Biology (Basel). 2025 Jun 18;14(6):715. doi: 10.3390/biology14060715 (PMC12190092; doi:10.3390/biology14060715)
Supplement: Supplementary file 1 [file biology-14-00715-s001.zip › Supplementary Materials S1: 16s-report of gut microbiota/Groups/diff_analysis/TaxaMarkers/Class.Groups.sig.boxplot.pdf]

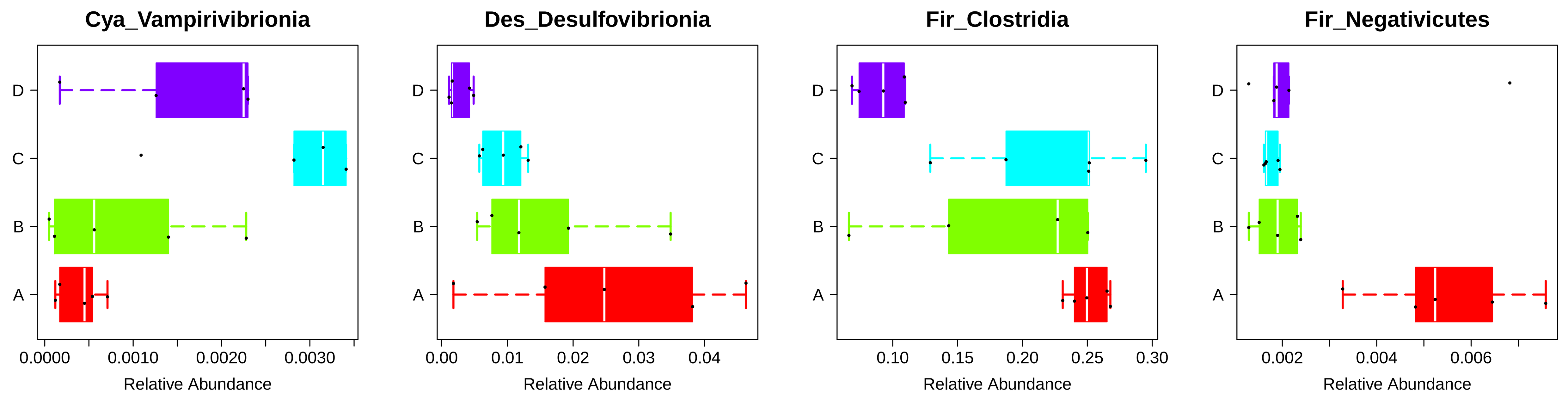
Cya_Vampirivibrionia
Des_Desulfovibrionia
Fir_Clostridia
Fir_Negativicutes
D
C
B
A
0.0000
0.0010
0.0020
0.0030
0.00
0.01
0.02
0.03
0.04
0.10
0.15
0.20
0.25
0.30
0.002
0.004
0.006
Relative Abundance
